# Beyond DNA damage response: Immunomodulatory attributes of CHEK2 in solid tumors

**DOI:** 10.18632/oncotarget.28740

**Published:** 2025-06-10

**Authors:** Helen Qian, Heba Ali, Vivekanudeep Karri, Justin T. Low, David M. Ashley, Amy B. Heimberger, Lucy A. Godley, Adam M. Sonabend, Crismita Dmello

**Affiliations:** ^1^Department of Neurological Surgery, Northwestern University Feinberg School of Medicine, Chicago, IL 60611, USA; ^2^Northwestern Medicine Malnati Brain Tumor Institute of the Lurie Comprehensive Cancer Center, Northwestern University Feinberg School of Medicine, Chicago, IL 60611, USA; ^3^College of Arts and Sciences, Cornell University, Ithaca, NY 14850, USA; ^4^Department of Zoology, Faculty of Science, Assiut University, Assiut, Egypt; ^5^Duke University School of Medicine, Duke University, Durham, NC 27708, USA; ^6^Division of Hematology/Oncology, Department of Medicine, Robert H. Lurie Comprehensive Cancer Center, Northwestern University, Chicago, IL 60611, USA

**Keywords:** *CHEK2*, immune checkpoint inhibitors, immunomodulation

## Abstract

The *CHEK2* gene serves a canonical role in the DNA damage response (DDR) pathway encoding the regulatory kinase CHK2 in the homologous recombination (HR) repair of double-strand breaks (DSB). Although *CHEK2* is traditionally considered a tumor suppressor gene, recent studies suggest additional functions. Across several cohort studies, *CHEK2* expression was negatively correlated with the efficacy of immune checkpoint inhibitors (ICI), which target the interaction between effector immune and tumor cells. This review explores two possible explanations for this observed phenomenon: the first relating to the canonical role of *CHEK2*, and the second introducing a novel role of the *CHEK2* gene in immunomodulation of the tumor microenvironment (TME). DDR mutations have been implicated in increased levels of tumor mutation burden (TMB), often manifesting as neoepitope expression on the tumor cell surface recognized by effector immune cells. As a result, impaired DNA repair due to *CHEK2* loss of function, either from germline deleterious variants or acquired mutations, results in the recruitment of CD8+ cytotoxic T-cells and subsequent efficacy of ICI treatment. However, functional loss of *CHEK2* may be directly involved in potentiating the immune response through canonical inflammatory and anti-tumor pathways, acting through the cGAS-STING pathway. Although the exact mechanism by which *CHEK2* modulates immune responses is still under investigation, combination therapy with CHEK1/2 inhibition and ICI immunotherapy has shown benefit in preclinical studies of several solid tumors.

## INTRODUCTION

Double-strand breaks (DSBs) in DNA are implicated in cancer development [1, 2]. DSB repair occurs through two main DNA damage response (DDR) pathways: homologous recombination (HR) and non-homologous end joining (NHEJ). HR utilizes an undamaged complementary DNA strand for the repair of a DSB, whereas NHEJ ligates the two broken DNA ends through random resection and addition of nucleotides [1]. Cell cycle CHEckpoint Kinase 2 (*CHEK2*) plays an integral role in the DDR pathway, and is thought to induce complete and accurate repair of DSBs through HR [3].

C*HEK2* encodes the CHK2 protein, an integral component of the HR pathway, which promotes the precise repair of DSBs. CHK2 is a protein kinase involved in the ataxia-telangiectasia mutated (ATM) serine/threonine kinase-CHK2-p53 DDR pathway [3]. The forkhead-associated (FHA) domain of CHK2 mediates the phosphorylation of CHK2 at its SQ/TQ cluster domain by ATM and the subsequent activation of BRCA1/2 and CDC25 family of effector proteins. Activation of the effector proteins induce p53 signaling, DNA repair (via BRCA 1/2), cell cycle regulation (via CDC25A), and/or apoptosis (via PML) [4]. By stabilizing p53 and inhibiting the mitotic promotor gene CDC25A through phosphorylation, CHK2 can induce either apoptosis or cell cycle arrest, acting canonically as a tumor suppressor [3, 4].

Loss of *CHEK2* function forces the cell to rely more error-prone DNA repair pathways, including NHEJ, which introduces DNA frameshift and missense mutations [1, 2]. *CHEK2* was initially identified as a cancer susceptibility gene in Li Fraumeni syndrome, first reported in 1999 [5]. The discovery of the c.1100delC truncating mutation provided evidence that *CHEK2* functions as a tumor suppressor, a role that remains widely supported in literature. Although *CHEK2*’s role was initially attributed to its interaction with ATM, studies suggest that *CHEK2* can also function independently of ATM in DSB repair [6].

The p.I157T mutation in the FHA domain was characterized shortly thereafter, disrupting protein-protein interactions and impairing downstream DDR effectors. Initially classified as a loss of function mutation, it now recognized as having a dominant-negative effect [4, 7]. The c.1100delC deletion and the I157T missense mutation are the two most prevalent germline *CHEK2* variants [8]. While this review focuses on *CHEK2* activity in the context of mRNA expression, it is important to note that *CHEK2* loss of function is also driven by defective phosphorylation and not just transcriptional alterations. Furthermore, following the initial discovery and characterization of *CHEK2* variants, these mutations have also been found in association with several cancers.

Breast cancer was the first malignancy to be directly linked to germline *CHEK2* variants, with multiple studies having shown that the c.1100delC mutation confers increased susceptibility to breast cancer [9–11]. Later studies found similarly elevated risk of lobular (OR = 4.17 95% CI = 2.89–6.03, *P* < 0.0001) and hereditary breast tumors in patients with *CHEK2* I157T germline variants (OR = 1.48, 95% CI = 1.16–1.89, *P* < 0.0001) [9]. Germline mutations of *CHEK2* have since been widely associated with several hereditary cancers [10, 12]. Meijers-Heijboer et al. 2003 identified the *CHEK2* 1100delC mutation to be associated in families with both hereditary breast and colorectal cancer [13]. Shortly after, *CHEK2* emerged as a multiorgan cancer susceptibility gene. In a study of 4008 Polish cancer patients, positive associations were identified between the *CHEK2* I157T missense mutation and thyroid, breast, and prostate cancer [14]. *CHEK2* deleterious variants are now among the most frequently detected alterations in multigene cancer panel testing [3]. In this review, we explore the effect of *CHEK2* alterations on the tumor immune microenvironment, both within and beyond the DDR pathway.

## *CHEK2* AS AN IMMUNOMODULATOR

### *CHEK2* expression is associated with tumor mutational burden (TMB) and neo-antigen load

Tumor mutational burden (TMB) measures the number of somatic mutations in tumor DNA and serves as potential predictor of response to immune checkpoint inhibitors (ICIs) [15], particularly in lung adenocarcinoma and melanoma - solid tumors that exhibit intrinsically high levels of somatic mutations [16]. Hugo et al. 2016 was the first to examine this phenomenon in a cohort of 38 melanoma samples, in which high levels of TMB conferred significantly improved patient survival and tumors from responding patients were enriched for BReast CAncer gene 2 (BRCA2) mutations. However, no significant association was drawn between response to anti-PD-1 and TMB [16].

Later studies were able to draw a positive correlation between TMB and the overall response rate to PD-1 (*P* < 0.001) [17]. Rizvi et al. 2015 first demonstrated tumor regression of non-small cell lung cancer (NSCLC) following pembrolizumab (a PD-1 inhibitor) treatment, particularly in tumor with high TMB expression, which led to an upregulation of neoantigen-specific CD8+ T cell responses [15]. A higher number of somatic mutations is associated with increased neoantigen production, which are then subsequently presented by major histocompatibility complexes (MHC) on the tumor cell surface and recognized by cytotoxic T-cells [18]. Carcinomas such as melanoma and NSCLC that are characterized by high numbers of somatic mutations show the strongest correlation between TMB rates and T-cell cytotoxicity as a high intrinsic number of mutations contributes to higher levels of TMB. Meta-analysis findings confirm this, with both small and non-small cell lung cancer exhibiting the highest overall survival (OS) in response to ICI treatment (HR 0.74, CI 95%) [19, 20].

However, this intrinsically high level of somatic mutations is not the case for the majority of cancer types. For example, glioblastoma lacks significant T-cell infiltration and therefore is largely unresponsive to ICI therapy [21]. Although neoantigen load has emerged as a predictor of overall survival [22, 23], patients with solid tumors other than melanoma and SCLC or NSCLC show lower prognostic favorability for ICI therapy, with response ranges between 15–30% [24]. It is this dilemma that introduces us to the role *CHEK2* may play in increasing the efficacy of ICI treatment. Similar to non-small cell lung cancer, increased neoantigen expression was positively correlated with overall survival and T-cell activation in breast and ovarian cancer [22, 25]. Notably, this increased neo-antigen load is significantly correlated with DSB repair pathway deficiencies. Given that *CHEK2* is directly involved in DSB repair, it is reasonable to hypothesize that *CHEK2* expression serves as a prognostic biomarker for ICI response. In ovarian cancer, T-cell activation was linked to *BRCA1/2* deficiencies, both effectors of the HR pathway. The number of CD3+ and CD8+ tumor-infiltrating lymphocytes was increased in HR pathway deficient cells, whereas *BRCA1/2* mutated tumors also showed increased infiltration of CD4^+^ cells [22]. Tumors with higher neo-antigen load were subsequently associated with higher overall survival rates. It is worth noting that this study was conducted in the context of HR mutations, including *CHEK2* mutants in the functionally HR depleted cohort. No significant difference in mutated and non-mutated *BRCA1/2* cells could be detected without loss of HR function. Therefore, it is suggested that *CHEK2* loss of function producing a deficient HR pathway may be responsible for higher TMB. Therefore, the assumption that the level of *CHEK2* expression is directly correlated with TMB load should be cautioned against – rather, it is the level of phosphorylated CHK2 protein that determines its effectivity. In other words, TMB load appears to be negatively correlated with the function of the *CHEK2*. Pan-cancer analysis of the TCGA database also suggests that the rate of *CHEK2* mutations, and not simply the level of *CHEK2* expression, are associated with higher frequencies of somatic mutations [26]. Although a growing correlation between CHEK2 loss of function and neo-antigen expression can be observed, further analysis of deleterious variants, knockout *CHEK2* cell lines and *in vivo* models is necessary to understand this relationship.

## *CHEK2* DEFICIENCY IS ASSOCIATED WITH RESPONSE TO IMMUNE CHECKPOINT INHIBITORS

PD-1 is widely expressed on the surfaces of cytotoxic T cells and binds to PD-L1 on somatic cells, an interaction that helps prevent autorecognition and regulate the immune response. PD-L1 expression on the surface of tumor cells promotes T-cell exhaustion and allows them to evade immune detection. ICIs, including anti-PD-1 and anti-PD-L1, disrupt this interaction, interrupting the immunosuppressive activity of solid tumor cells and promoting T-cell cytotoxicity [27].

As previously described, the canonical function of *CHEK2* in the DDR pathway may have counterintuitive implications. Similar to neoantigen load, an increase in PD-L1 expression in ovarian cancer has been correlated with *CHEK2* expression [22]. Intraepithelial and peri-tumor lymphocytes in the ovarian TME also showed higher PD-1 levels [22]. This suggests that PD-1/PD-L1 inhibitors may be more effective in *CHEK2*-deficient tumors, an observation which has been noted in both murine and pan-cancer studies. In *CHEK2* deficient tumor lines treated with PD-1 inhibitor, tumors showed significant regression in volume and increased CD8+ T-cell infiltration [26]. This was particularly observed with IFNγ^+^ CD8^+^ and granzyme B^+^ CD8^+^ T cell populations. Interestingly, single cell RNA-sequencing of ICI treated *CHEK2* knockout cells revealed a number of differentially expressed genes (DEGs) involved in inflammatory, cytokine, and innate/adaptive immunity pathways [26], in comparison to untreated *CHEK2*-deficient cell lines. This observation suggests that *CHEK2* exerts an influence on innate immune processes, although its exact mechanism remains unclear. It appears that *CHEK2* presence may play an active immunosuppressive role, with *CHEK2* loss of function inducing proinflammatory cytokine pathways.

Notably, treatment of solid tumors with strong immunosuppressive properties showed significant therapeutic benefit following genetically induced *CHEK2* deficiencies in murine models of colorectal carcinoma and melanoma. These models exhibited marked tumor regression following ICI treatment [26]. For tumors traditionally unresponsive to ICI treatment, combination therapy with *CHEK2* intrinsic deficiency may prove beneficial to overall survival. Indeed, ICI treatment of melanoma, transitional cell carcinoma (TCC), and renal cell carcinoma patients with DDR germline mutations showed favorable results [28, 29]. Patient tumors harboring DDR mutations, including 1100delC *CHEK2* mutants and/or *BRCA1/2* deleterious variants, showed higher percentages of tumor shrinkage (ORR = 86%) and longer overall survival (PFS = 30 months). Although this study included only seven tumors, the wide range of solid tumors that displayed favorable responses to ICI therapy suggests that CHEK2 may serve as a biomarker of response to ICI.

**Figure 1:**
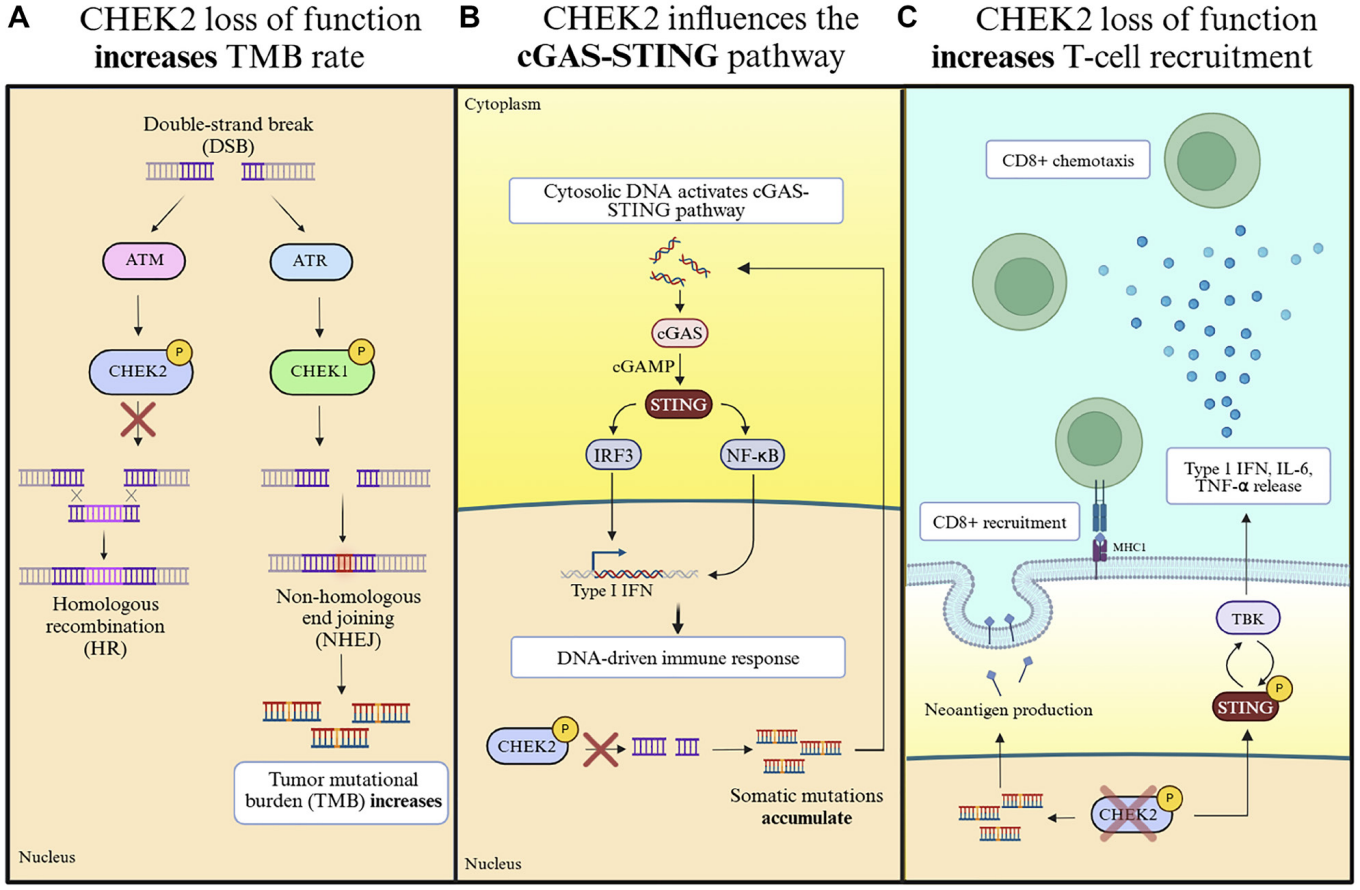
The proposed effect of CHEK2 loss of function on cytotoxic T-cell recruitment at different organizational levels. (**A**) Double stranded breaks (DSB) are resolved through either homologous recombination or the non-homologous end joining pathway. In the loss of CHEK2 function scenario, the functionality of the HR pathway is reduced, and the ability to resolve double stranded breaks fully and accurately, is diminished. Subsequently, the DNA damage response pathway attempts to resolve the DSB through the NHEJ pathway, which is less accurate and leads to the accumulation of somatic mutations. (**B**) The accumulation of somatic mutations produces fragments of DNA that exit the nucleus through vesicles. When DNA is released from these vesicles, cGAS recognizes the presence of cytosolic DNA and activates the downstream effectors of the cGAS-STING pathway. (**C**) The secondary mechanism of immune cell recruitment including CD8 T cell infiltration because of CHEK2 deficiency maybe through the downstream signaling cascade leading to the production of Type I Interferon and chemotaxis inducing cytokines following cGAS-STING pathway activation.

With *CHEK2* and *BRCA1/2* germline variants showing similar positive responses to ICI treatment, it appears these results may partly be due to loss of function in the DDR pathway. Figure 1 summarizes three known mechanisms by which inhibition of *CHEK2* in tumor cells promotes an immune response. Taken together, *CHEK2* expression has the potential to predict resistance or non-response to immunotherapy.

## ALTERNATIVE ACTIVATION OF THE IMMUNE RESPONSE IS ASSOCIATED WITH THE LOSS OF *CHEK2* FUNCTION

Although it appears that much of *CHEK2’s* immunomodulating capabilities arise from its role in the DDR pathway, new evidence suggests that CHEK2 may play a novel role in directly altering the immune tumor microenvironment.

In renal cell carcinoma, high *CHEK2* expression has been correlated with lower overall survival (OS) and an overall poor prognosis. *CHEK2* expression was found to be positively correlated with immune checkpoint marker expression and regulatory, immunosuppressive T-cell (Treg) infiltration, but negatively correlated with pro-inflammatory M1 macrophages, even without ICI therapy. The upregulation of inhibitory immune checkpoints and Treg cells facilitate tumor immune evasion and suppression, while the absence of pro-inflammatory, anti-tumor M1 macrophages similarly aids in tumor proliferation unadulterated by the immune system. Lowered expression of *CHEK2* was also correlated with low TIDE scores, a combination of T-cell dysfunction and T-cell exclusion gene signatures, indicating a superior intrinsic immune response. Additionally, tumors with low and high *CHEK2* expression groups showed different levels of antigen presenting cell (APC) recruitment in the TME and IFN response [30]. Xu et al., 2024 have also previously explored the differentially expressed genes in innate immune pathways of CHEK2-KO lines treated with ICI [26], further suggesting an alternative, immune-specific pathway in which the *CHEK2* gene may play a role.

*CHEK2* deficiency in gliomas points to this possible alternative pathway. Loss of MHC expression, immune suppressive cytokines (TGF-B, IL-10, PGE2), and the expression of PD-L1 in patients with glioblastoma (GBM) contribute to a TME enriched with immunosuppressive factors, rendering these individuals resistant to ICI therapy [31]. This study further supports the tumor-intrinsic origin of GBM associated immunosuppression. Consistent with this, our findings showed that low CHEK2 expression in glioma tumor cells was correlated with higher T-cell infiltration and upregulation of the antigen presentation pathway. Tumor cells with low *CHEK2* displayed enrichment of type I interferon in the TME and a subsequently higher PD-L1 expression induced by IFN-γ [32]. This was accompanied by STING pathway activation in response to *CHEK2* depletion, indicating that the *CHEK2* kinase plays an inhibitory role in the type I interferon (IFN) release pathway. Type I IFN is known to induce T-cell infiltration [33], making this an exciting discovery of the possible immunotherapeutic benefits *CHEK2* inhibition may offer. STING activation and the subsequent transcription of IFN-regulatory genes (IRFs) depend in part on the recognition of cytosolic DNA [24, 34]. Upon recognition, cGAS catalyzes the conversion of GTP and ATP into cGAMP (2’,3’-cyclilc guanosine monophosphate-adenosine monophosphate). cGAMP then stimulates the STING protein, which recruits Tank binding kinase (TBK1) to auto-phosphorylate and phosphorylate IRF 3, 5, and 7 [34]. The ensuing type I IFN release contributes to the recruitment of cytotoxic T-cells.

*CHEK2* loss of function may cause DNA fragments from unrepaired DSBs to enter the cytosol and activate type I IFN signaling (Figure 1). Indeed, growing evidence exists to connect the DDR pathway to cGas-STING pathway activation. A recent *in-vitro* study on pediatric gliomas with the *H3.3-G34R/V* mutation demonstrates that this mutation downregulates DNA repair pathways, as it co-occurs with *TP53* and *ATRX* mutations, which are critical DDR effectors. The downregulation of DNA repair leads to the accumulation of extrachromosomal DNA, activating the STING-cGAS pathway. This observation is further supported by the therapeutic efficacy of STING-agonists in *H3.3-G34R/V* mutant gliomas [35]. Additionally, in ATM-deficient cells, treatment with an ATR inhibitor (ceralasertib) and a cytotoxic agent (PBD SG-3199) showed elevated levels of interferon signaling gene (ISG) expression, both before and after treatment. The subsequent upregulation of IFN-α and IFN-β release, mediated by the cGAS-STING pathway, enhanced the recruitment of dendritic cells (DCs) in the TME, which in turn stimulated CD8+ T cell responses [36]. Interestingly, the same study suggests that ATM may directly interact with the STING protein, though this interaction is not yet well understood. CHK1 inhibitors have also been found to increase PD-L1 expression on tumor cell surfaces, potentiating response to immune checkpoint blockade [37]. Considering the connection between the cGAS-STING pathway and other DDR effectors, it is plausible to postulate that *CHEK2* affects this pathway in the same way. However, the magnitude of impact of CHK2, as opposed to other DDR players, in triggering the type I IFN pathway has not yet been determined. Nonetheless, there is compelling preclinical evidence to suggest a combination therapy with CHK2 inhibition and PD-1/PD-L1 inhibitors can exhibit an efficacious response.

## USE OF *CHEK2* INHIBITION IN COMBINATION THERAPEUTICS

Several ongoing and completed clinical trials involve the *CHEK2* inhibitors prexasertib and AZD7762 (Table 1). Prexasertib is an ATP-selective inhibitor of CHEK1/2 currently being evaluated across numerous clinical trials, both as a monotherapy and in combination with PARP inhibitors, chemotherapy agents, and platinum-based radiation [38]. AZD7762 is another CHEK1/2 inhibitor which underwent early-stage evaluation, with 3 clinical trials evaluating its tolerability with and without gemcitabine. However, its use was terminated over concerns of unpredictable cardiac toxicity [39]. In a Phase I clinical study, prexasertib was tested in combination with the PD-L1 inhibitor pembrolizumab, showing clinical benefit in 8 out of 14 patients with recurrent ovarian cancer, including patients with significant partial responses. Patients with the longest-lasting responses displayed higher levels of T-cell recruiting cytokine signatures, including increases in IL-2, IL-7, and IL-17. Additionally, T-cell activation was increased, whereas T-reg and CD8+ NKT immunosuppressive cell populations decreased after a single dose of prexasertib. The initial results from this Phase I clinical trial support the immunomodulatory role of *CHEK2* expression and even suggest *CHEK2* potentiates immunosuppression. These promising results should be taken with caution, as prexasertib is a more potent inhibitor of *CHEK1*. Therefore, these results cannot be attributed to *CHEK2* alone. [40]. Nonetheless, it also suggests that dual inhibition of CHEK*1* and *CHEK2* may enhance immune activation.

**Table 1:** Clinical trials involving CHEK2 inhibitors in combination with ICPI treatment

ID	Cancer	*n*	*CHK2* inhibitor	Clinical trial stage	Target
NCT03495323	High grade serous ovarian cancer	17	Prexasertib	I	Cytotoxic T-cell recruitment w/ICPI/CHK1/2i combination therapy
NCT03414047	Refractory/Recurrent Ovarian Cancer	172	Prexasertib	II	Safety of prexasertib in platinum-resistant ovarian cancer
NCT04032080	Metastatic Triple-negative Breast Cancer	10	Prexasertib	II	Inhibit NHEJ DNA repair
NCT02778126	Advanced cancer	6	Prexasertib	I	Evaluate prexasertib pharmacokinetics
NCT02514603	Advanced cancer	12	Prexasertib	I	Tolerability of prexasertib
NCT02808650	Refractory/Recurrent Solid Tumors	30	Prexasertib	I	Pharmacokinetics in pediatric patients, CHK1/2/TP53 evaluation
NCT03057145	Advanced solid tumors	29	Prexasertib	I	CHK1i/PARPi combination therapy
NCT02873975	Solid Tumor	27	Prexasertib	II	Prexasertib efficacy in HR deficient tumor
NCT02735980	SCLC	133	Prexasertib	II	Pharmacokinetics in end-stage SCLC
NCT02124148	Advanced Cancer	167	Prexasertib	Ib	Safety of prexasertib/chemotherapy combination therapy
NCT02860780	Advanced/Metastatic cancer	9	Prexasertib	I	Safety of prexasertib/ralimetinib combination therapy
NCT02555644	Head and neck cancer	70	Prexasertib	I	Safety of prexasertib w/chemotherapy/radiation
NCT01115790	Head and neck SCC, advanced solid tumor	150	Prexasertib	I	Safety/toxicity of prexasertib
NCT03735446	Refractory AML, MDS	2	Prexasertib	I	Targeted combination therapy
NCT04095221	Desmoplastic Small Round Cell Tumor, Rhabdomyosarcoma	21	Prexasertib	I, II	Dose safety of prexasertib in combination therapy
NCT02649764	AML, MDS	15	Prexasertib	I	Dose safety, side effects of combination therapy
NCT04023669	Medulloblastoma	21	Prexasertib	I	Prexasertib/DNA damaging agent combination therapy
NCT02203513	High grade serous ovarian cancer, triple-negative breast cancer	111	Prexasertib	II	Determine tumor shrinkage with prexasertib
NCT00937664	Advanced solid tumors	24	AZD7762^*^	I	Dose escalation/tolerability of combination therapy
NCT00473616	Solid tumor	60	AZD7762^*^	I	Dose escalation of AZD7762
NCT00413686	Solid tumor	42	AZD7762^*^	I	Safety, tolerability, biomarkers of AZD7762 with/without gemcitabine

AZD7762 (trials indicated with an asterisk) discontinued in 2011 due to concerns over cardiac toxicities.

## CONCLUSIONS

In this review, we examined the role of *CHEK2* in the context of its immunomodulatory capabilities, as the canonical role of *CHEK2* in the DDR pathway is already well known. This paper has curated the effects of *CHEK2* loss of function on the immune response against solid tumors. Growing evidence suggests that the loss of *CHEK2* function may disrupt accurate double-stranded repair, contributing to the accumulation of somatic mutations and subsequent expression of neoantigens. Additionally, *CHEK2* loss appears to activate the cGAS-STING pathway, thereby stimulating the effector immune cell population and driving inflammatory responses. Whether this observed phenomenon is a consequence of *CHEK2*’s role in the DDR pathway, or results from a novel interaction between CHK2 and cGAS-STING effectors, remains to be investigated.

In this context, CHK2 may be a potential target in increasing the efficacy of existing ICI therapies, which have shown low efficacy rates in many solid tumors. Currently, inhibitors that target CHK2 kinase activity are available. However, the non-catalytic function of CHK2 remains unexplored, not to mention the unpredictability of CHK2 inhibition in combination with ICI therapy. Our group is actively investigating the non-canonical immunomodulatory functions of CHK2 and working to develop a therapeutic agent that targets multiple CHK2 activities.

## Abbreviations

ATM: Ataxia-Telangiectasia Mutated kinase
BRCA: BReast CAncer gene
*CHEK1*: CHeckpoint kinase 1
*CHEK2*: Human CHeckpoint kinase 2 gene
CHK2: Human Checkpoint kinase 2 protein
DDR: DNA Damage Response
DSB: Double-Stranded Break
FHA: Forkhead Association domain
HR: Homologous Recombination
ICPI: Immune Checkpoint Inhibitor
ORR: Overall Response Rate
OS: Overall Survival
PD-1: Programmed Death receptor
PD-L1: Programmed Death Ligand
PR: Partial Response
PV: Pathogenic Variant
TME: Tumor Microenvironment
VUS: Variant of Unknown Significance
